# Entire functions sharing a small function with their two difference operators

**DOI:** 10.1186/s13662-017-1281-4

**Published:** 2017-08-01

**Authors:** Feng Lü, Yanfeng Wang, Junfeng Xu

**Affiliations:** 1 College of Science, China University of Petroleum, Qingdao, Shandong 266580 P.R. China; 2 Department of Mathematics, Wuyi University, Jiangmen, Guangdong 529020 P.R. China

**Keywords:** 30D35, 30D30, 39A10, uniqueness, entire functions, difference operators, Nevanlinna theory

## Abstract

In this article, we deduce a uniqueness result of entire functions that share a small entire function with their two difference operators, generalizing some previous theorems of (Farissi et al. in Complex Anal. Oper. Theory 10:1317-1327, 2015, Theorem 1.1) and (Chen and Li in Adv. Differ. Equ. 2014:311, 2014, Theorem 1.1) by omitting the assumption that the shared small entire function is periodic.

## Introduction and main result

Nevanlinna theory of value distributions is concerned with the density of points where a meromorphic function takes a certain value in the complex plane. Nowadays, there has been recent interest in connections between the Nevanlinna theory and the difference operator. In addition, many papers have been devoted to the investigation of the uniqueness problems related to meromorphic functions and their shifts or their difference operators and one got a lot of results (see, *e.g.*, [3–8]).

In order to state the main result, we give the following definition. For a meromorphic function $f(z)$, we define its shift by $f_{c}=f(z+c)$ and its difference operators by

$$\begin{aligned} &\Delta_{c}f(z)=f(z+c)-f(z), \\ &\Delta^{n}_{c}f(z)= \Delta^{n-1}_{c} \bigl(\Delta_{c}f(z) \bigr), \quad n \in \mathbb{N}, n\geq 2. \end{aligned}$$

A meromorphic function $a(z)$ is said to be a small function with respect to $f(z)$ if and only if $T(r,a)=S(r,f)$, where $S(r,f)=o(T(r,f))$, as $r\rightarrow \infty $ outside of a possible exceptional set of finite logarithmic measure. Denote the set of all the small functions of $f(z)$ by $S(f)$. Let $f(z)$ and $g(z)$ be two meromorphic functions and let $a(z)$ be a small entire function of $f(z)$ and $g(z)$. We say that $f(z)$ and $g(z)$ share $a(z)$ IM, provided that $f(z)-a(z)$ and $g(z)-a(z)$ have the same zeros ignoring multiplicities. Similarly, we say that $f(z)$ and $g(z)$ share $a(z)$ CM, provided that $f(z)-a(z)$ and $g(z)-a(z)$ have the same zeros counting multiplicities.

Recently, Chen *et al.* [2, 9] investigated two uniqueness problems on entire functions that share a small periodic entire function with their two difference operators as follows.

### Theorem A

see [9], Theorem 1.1

*Let* $f(z)$ *be a nonconstant entire function of finite order*, *let* $a(z)(\not \equiv 0)\in S(f)$ *be a periodic entire function with period* *c*. *If* $f(z)$, $\Delta_{c}f(z)$, $\Delta^{2}_{c}f(z)$ *share* $a(z)$ *CM*, *then* $\Delta^{2}_{c}f(z)\equiv \Delta_{c}f(z)$.

### Theorem B

see [2], Theorem 1.2

*Let* $f(z)$ *be a nonconstant entire function of finite order*. *If* $f(z)$, $\Delta_{c}f(z)$, $\Delta^{2}_{c}f(z)$ *share* 0 *CM*, *then* $\Delta^{2}_{c}f(z)\equiv C \Delta_{c}f(z)$, *where* *C* *is a nonzero constant*.

In 2015, El Farissi, Latreuch and Asiri further studied the above problem and obtained

### Theorem C

see [1], Theorem 1.1

*Let* $f(z)$ *be a nonconstant entire function of finite order*, *let* $a(z)(\not \equiv 0) \in S(f)$ *be a periodic entire function with period* *c*. *If* $f(z)$, $\Delta_{c}f(z)$, $\Delta^{2}_{c}f(z)$ *share* $a(z)$ *CM*, *then* $f(z)\equiv \Delta_{c} f(z)$.

### Remark 1

It is necessary to point out that Theorems A and B have been generalized from $\Delta^{2}_{c}f(z)$ to $\Delta^{n}_{c}f(z)$ by Chen, Chen and Li in [9]. There are also some interesting results related the above theorems (see, *e.g.*, [6, 10]).

In the previous results, we find that the shared small function $a(z)$ is a periodic function with period *c*. So, it is natural to ask what will happen if the periodic condition of $a(z)$ is omitted. In this paper, we focus on this problem and we obtain the following result.

### Theorem 1

*Let* $f(z)$ *be a nonconstant entire function of finite order*, *and let* $a(z)\in S(f)$ *be an entire function*. *If* $f(z)$, $\Delta_{c}f(z)$, $\Delta^{2}_{c}f(z)$ *share* $a(z)$ *CM*, *then one of the following assertions holds*:

(i) *If* $\Delta_{c}a(z)\equiv a(z)$, *then* $\Delta^{2}_{c}f(z)-a(z)=C( \Delta_{c}f(z)-a(z))$, *where* *C* *is a nonzero constant*.
(ii) *If* $\Delta_{c} a(z)\not \equiv a(z)$, *then* $\Delta_{c}f(z)=f(z)$ *or* $\Delta^{2}_{c}f(z)-a(z)=e^{\gamma }(\Delta_{c}f(z)-a(z))$, *where* *γ* *is a polynomial with* $\deg \gamma < \rho (a)$.

### Remark 2

We point out that Theorem 1 is a generalization of the previous theorems.

If $a(z)\equiv 0$, then $\Delta_{c}a(z)=a(z)$. Then it follows from (i) of Theorem 1 that $\Delta^{2}_{c}f(z)=C \Delta_{c}f(z)$, where *C* is a nonzero constant.

If $a(z)\not \equiv 0$ is a periodic function with period *c*, then $\Delta_{c} a(z)\not \equiv a(z)$. It follows from (ii) of Theorem 1 that $\Delta_{c}f(z)=f(z)$ or $\Delta^{2}_{c}f(z)= \Delta_{c}f(z)$. Furthermore, by Theorem C we can deduce that $\Delta_{c}f(z)=f(z)$.

As an application of Theorem 1, we can obtain an interesting result, where $a(z)$ is a slow growth small function.

### Theorem 2

*Let* $f(z)$ *be a nonconstant entire function of finite order*, *and let* $a(z)(\not \equiv 0)\in S(f)$ *be an entire function with* $\rho (a)<1$. *If* $f(z)$, $\Delta_{c}f(z)$, $\Delta^{2} _{c}f(z)$ *share* $a(z)$ *CM*, *then* $\Delta_{c}f(z)=f(z)$.

For convenience of the reader, we list here some notations. For a meromophic function *f*, we use the basic notations of the Nevanlinna theory of meromorphic functions such as $T(r,f)$, $m(r,f)$, $N(r,f)$ and $\overline{N}(r,f)$ as explained in [11–13].

## Some lemmas

In this section, we state some results that we employ in our proofs.

### Lemma 2.1

[4], Theorem 2.1

*Let* $c\in \mathbb{C}, n\in \mathbb{N}$, *and let* *f* *be a meromorphic function with a finite order*. *Then for all small periodic functions* $a(z)\in S(f)$

$$ m \biggl(r,\frac{\Delta_{c}^{n} f(z)}{f(z)-a(z)} \biggr)=S(r,f), $$

*where* $S(r, f )= o(T (r, f ))$ *for all* *r* *outside of a possible exceptional set* *E* *with finite logarithmic measure*.

### Lemma 2.2

[14], Lemma 3.3

*Let* *g* *be a nonconstant meromorphic function in the plane of order less than* 1, *and let* $h>0$. *Then there exists a* *ϵ*-*set* *E* *such that*

$$ \frac{g(z+\eta )}{g(z)}\rightarrow 1,\quad \textit{as } z\rightarrow \infty \textit{ in } \mathbb{C}\backslash E, $$

*uniformly in* *η* *for* $\vert \eta \vert < h$.

Lemma 2.2 plays an important role in the proof of Theorem 2.

## Proof of Theorem 1

Note that $f(z)$ is a nonconstant entire function of finite order. Then $\Delta_{c}f(z)$ and $\Delta^{2}_{c}f(z)$ are also two entire functions of finite order.

Set $g(z)=f(z)-a(z)$. Then

$$ \Delta_{c} g(z)=\Delta_{c} f(z)-\Delta_{c}a(z), \qquad \Delta^{2}_{c} g(z)= \Delta^{2}_{c} f(z)-\Delta^{2}_{c} a(z). $$

Since $f(z)$, $\Delta_{c}f(z)$, $\Delta^{2}_{c}f(z)$ share $a(z)$ CM, we have

1 $$\begin{aligned}& \frac{\Delta_{c}f(z)-a(z)}{f(z)-a(z)}=\frac{\Delta_{c} g(z)+\Delta _{c} a(z)-a(z)}{g(z)}=e^{P(z)}, \end{aligned}$$

2 $$\begin{aligned}& \frac{\Delta^{2}_{c}f(z)-a(z)}{f(z)-a(z)}=\frac{\Delta^{2}_{c} g(z)+ \Delta^{2}_{c} a(z)-a(z)}{g(z)}=e^{Q(z)}, \end{aligned}$$

where $P(z)$ and $Q(z)$ are two polynomials.

Suppose that $\Delta_{c} a(z)\equiv a(z)$. Obviously, we can get $\Delta^{2}_{c} a(z)\equiv a(z)$. It is clear that $g(z)$, $\Delta _{c} g(z)$, and $\Delta^{2}_{c} g(z)$ share 0 CM. By Theorem B, we can obtain $\Delta^{2}_{c} g(z)\equiv C\Delta_{c}g(z)$, where *C* is a nonconstant. So $\Delta^{2}_{c} f(z)-\Delta^{2}_{c}a(z)\equiv C(\Delta _{c}f(z)-\Delta_{c}a(z))$. That is, $\Delta^{2}_{c} f(z)-a(z)\equiv C( \Delta_{c}f(z)-a(z))$.

In the following, we assume that $\Delta_{c} a(z)\not \equiv a(z)$. We consider into two cases.

*Case 1.* $\Delta^{2}_{c} a(z)\not \equiv a(z)$.

Set

3 $$\begin{aligned} \varphi (z) &=\frac{(\Delta_{c} a(z)-a(z))(\Delta^{2}_{c}f(z)-\Delta ^{2}_{c}a(z))-(\Delta^{2}_{c} a(z)-a(z))(\Delta_{c}f(z)-\Delta_{c}a(z))}{f(z)-a(z)} \end{aligned}$$

4 $$\begin{aligned} &=\frac{(\Delta_{c} a(z)-a(z))(\Delta^{2}_{c}f(z)-a(z))-(\Delta^{2} _{c} a(z)-a(z))(\Delta_{c}f(z)-a(z))}{f(z)-a(z)}. \end{aligned}$$

From (1) and (2), we can rewrite the above function as

5 $$ \varphi (z)= \bigl(\Delta_{c} a(z)-a(z) \bigr)e^{Q(z)}- \bigl(\Delta^{2}_{c} a(z)-a(z) \bigr)e ^{P(z)}, $$

which implies that *φ* is an entire function.

By (4) and Lemma 2.1, we deduce that $\varphi (z)\in S(f)$.

*Subcase 1.1.* We assume that $\varphi \not \equiv 0$. Rewrite (5) as

$$ 1+\frac{(\Delta^{2}_{c} a(z)-a(z)) e^{P(z)}}{\varphi (z)}= \bigl(\Delta_{c} a(z)-a(z) \bigr)\cdot \frac{e^{Q(z)}}{\varphi (z)}. $$

By the second main theorem, we deduce

$$\begin{aligned} &T \biggl(r, \bigl(\Delta^{2}_{c}a(z)-a(z) \bigr) \frac{e^{P}}{\varphi } \biggr) \\ &\quad \leq \overline{N} \biggl(r,\frac{1}{(\Delta^{2}_{c}a(z)-a(z))\frac{e ^{P}}{\varphi }} \biggr)+\overline{N} \biggl(r,\frac{1}{(\Delta^{2}_{c} a(z)-a(z))\frac{e ^{P}}{\varphi }+1} \biggr) \\ &\quad \quad {}+\overline{N} \biggl(r, \bigl(\Delta^{2}_{c}a(z)-a(z) \bigr)\frac{e^{P}}{\varphi } \biggr)+S(r,f) \\ & \quad \leq \overline{N} \biggl(r, \frac{1}{\frac{e^{P}}{\varphi }} \biggr)+ \overline{N} \biggl(r, \frac{1}{(\Delta_{c} a(z)-a(z))\cdot \frac{e^{Q}}{ \varphi }} \biggr)+\overline{N} \biggl(r, \frac{e^{P}}{\varphi } \biggr)+S(r,f) \\ &\quad \leq 2\overline{N}(r,\varphi )+N \biggl(r,\frac{1}{\varphi } \biggr)+S(r,f) \\ &\quad =S(r,f). \end{aligned}$$

Hence

$$ T\bigl(r,e^{P}\bigr)\leq T\biggl(r,\bigl(\Delta^{2}_{c} a(z)-a(z)\bigr)\frac{e^{P}}{\varphi }\biggr)+T\biggl(r,\frac{ \varphi }{(\Delta^{2}_{c} a(z)-a(z))}\biggr)\leq S(r,f). $$

Similarly, we get $T(r,e^{Q})=S(r,f)$.

Rewrite equation (1) as

6 $$ \Delta_{c} f(z)-a(z)= \bigl(f(z)-a(z) \bigr)e^{P(z)}=f(z+c)-f(z)-a(z), $$

which implies that $f(z+c)=f(z)(e^{P(z)}+1)+a(z)(1-e^{P(z)})$. Then we deduce

7 $$\begin{aligned} \Delta^{2}_{c} f(z) &=f(z+2c)-2f(z+c)+f(z) \\ &=f(z) \bigl[ \bigl(1+e^{P(z+c)} \bigr) \bigl(1+e^{P(z)} \bigr)-2 \bigl(1+e^{P(z)} \bigr)+1 \bigr]+a(z+c) \bigl(1-e^{P(z+c)} \bigr) \\ &\quad {}+a(z) \bigl[ \bigl(1+e^{P(z+c)} \bigr) \bigl(1-e^{P(z)} \bigr)-2 \bigl(1-e^{P(z)} \bigr) \bigr]. \end{aligned}$$

By (2), we have

8 $$ \Delta^{2}_{c} f(z)=f(z)e^{Q(z)}+a(z) \bigl(1-e^{Q(z)} \bigr). $$

Combining equations (7) and (8) yields

9 $$\begin{aligned}& f(z) \bigl[ \bigl(1+e^{P(z+c)} \bigr) \bigl(1+e^{P(z)} \bigr)-2 \bigl(1+e^{P(z)} \bigr)+1 \bigr]=f(z)e^{Q(z)}, \end{aligned}$$

10 $$\begin{aligned}& a(z+c) \bigl(1-e^{P(z+c)} \bigr)+a(z) \bigl[ \bigl(1+e^{P(z+c)} \bigr) \bigl(1-e^{P(z)} \bigr)-2 \bigl(1-e^{P(z)} \bigr) \bigr]=a(z) \bigl(1-e ^{Q(z)} \bigr). \end{aligned}$$

From (9), we have

11 $$ e^{P(z)} \bigl(e^{P(z+c)}+e^{P(z+c)-P(z)}-1 \bigr)=e^{Q(z)}. $$

Suppose that $e^{P(z)}$ is a nonconstant function. Denote $\eta =e ^{P(z+c)-P(z)}-1$. Obviously, *η* is a small function of $e^{P(z)}$. It follows from (11) that $e^{P(z+c)}+\eta $ have no zeros. Note that $e^{P(z+c)}$ has only one Picard value, say 0. Then $\eta \equiv 0$, which implies $e^{P(z+c)-P(z)}=1$. Again by (11), we have

$$ \bigl(e^{P(z)} \bigr)^{2}=e^{Q(z)}. $$

If $e^{P(z)}$ is a constant, then $e^{P(z+c)}=e^{P(z)}$. By (11) we also get $(e^{P(z)})^{2}=e^{Q(z)}$.

Furthermore, it follows from (10), $e^{P(z+c)}=e^{P(z)}$ and $(e^{P(z)})^{2}=e^{Q(z)}$ that

$$\begin{aligned}& \bigl(a(z+c)-2a(z) \bigr) \bigl(1-e^{P(z)} \bigr)=0, \\& \bigl(\Delta_{c}a(z)-a(z) \bigr) \bigl(1-e^{P(z)} \bigr)=0. \end{aligned}$$

Note that $a(z)\neq \Delta_{c}a(z)$, then $e^{P(z)}\equiv 1$, so $e^{Q(z)}\equiv 1$. Hence, we obtain $\Delta_{c}f(z)=f(z)$.

*Subcase 1.2.* We assume that $\varphi (z)\equiv 0$. Then it follows from (3) and (5) that

$$ \frac{\Delta^{2}_{c}f(z)-\Delta^{2}_{c}a(z)}{\Delta_{c}f(z)-\Delta _{c}a(z)}=\frac{\Delta^{2}_{c} a(z)-a(z)}{\Delta_{c} a(z)-a(z)}=e^{Q-P}=e ^{\gamma }, $$

where $\gamma =Q-P$ is a polynomial and $\deg \gamma < \rho (a)$. From this, we also have $\Delta^{2}_{c}f(z)-a(z)=e^{\gamma }(\Delta_{c}f(z)-a(z))$.

*Case 2.* $\Delta^{2}_{c}a(z)\equiv a(z)$.

By (2) and Lemma 2.1, we get $e^{Q}\in S(f)$.

Rewrite (1) and (2) as

12 $$\begin{aligned}& \Delta_{c} g(z)=g(z)e^{P(z)}+a(z)- \Delta_{c} a(z), \end{aligned}$$

13 $$\begin{aligned}& \Delta^{2}_{c} g(z)=g(z)e^{Q(z)}. \end{aligned}$$

Then

14 $$ e^{Q(z)}g(z)=e^{P(z+c)}g(z+c)-e^{P(z)}g(z)+ \Delta_{c} a(z)-a(z). $$

Rewrite (12) as $g(z+c)=g(z)[1+e^{P(z)}]+a(z)-\Delta_{c} a(z)$.

Now, substitute the form of $g(z+c)$ into (14) yields

15 $$ \bigl(e^{P(z+c)}e^{P(z)}+e^{P(z+c)}-e^{P(z)}-e^{Q(z)} \bigr)g(z)= \bigl(\Delta a(z)-a(z) \bigr) \bigl(e ^{P(z+c)}-1 \bigr). $$

Suppose that $n=\deg P\leq \deg Q$. Since $e^{Q}\in S(f)$, we have $e^{P}\in S(f)$.

Similar to the discussion of Subcase 1.1, we obtain $\Delta_{c}f(z)=f(z)$.

Now, we assume that $\deg P > \deg Q$. Suppose that

$$\begin{aligned} P(z)=a_{n}z^{n}+a_{n-1}z^{n-1}+\cdots +a_{0},\quad a_{n}\neq 0. \end{aligned}$$

Set $h=e^{a_{n}z^{n}}$. Then $e^{P(z)}=\beta_{1}h$, $e^{P(z+c)}=\beta _{2}h$, where $\beta_{1}$, $\beta_{2}$ are two small functions of *h*.

Note that $\deg P > \deg Q$, so $e^{Q}$ is also a small function of *h*.

From (15), we have

16 $$ \frac{\Delta_{c} a(z)-a(z)}{g(z)}=\frac{e^{P(z)}e^{P(z+c)}+e^{P(z+c)}-e ^{P(z)}-e^{Q(z)}}{e^{P(z+c)}-1}. $$

We claim that

$$ \frac{e^{P(z)}e^{P(z+c)}+e^{P(z+c)}-e^{P(z)}-e^{Q(z)}}{e^{P(z+c)}-1} $$

is irreducible except the factor which is a small function of *h*.

Suppose that $z_{0}$ is a common zero of $e^{P(z+c)}-1$ and $e^{P(z)}e^{P(z+c)}+e^{P(z+c)}-e^{P(z)}-e^{Q(z)}$. It is easy to deduce that $e^{Q(z_{0})}=1$. Note that $e^{Q(z)}-1\in S(h)$. Thus, the claim holds.

Rewrite (16) as

17 $$ \frac{\Delta_{c} a(z)-a(z)}{g(z)}= \frac{\beta_{1}\beta_{2}h^{2}+( \beta_{2}-\beta_{1}) h-e^{Q(z)}}{\beta_{2}h-1}. $$

Denote $H=\beta_{1}\beta_{2}h^{2}+(\beta_{2}-\beta_{1}) h-e^{Q(z)}= \alpha_{1}h^{2}+\alpha_{2}h+\alpha_{3}$, where $\alpha_{j}\in S(h)$, $j=1, 2, 3$. By the above equation, we see that $T(r,g)=O(T(r,h))$, which implies that $a\in S(h)$. Next we can prove that

$$ \overline{N} \biggl(r,\frac{1}{H} \biggr)\leq N \biggl(r, \frac{1}{\Delta_{c} a(z)-a(z)} \biggr)+S(r,h). $$

In fact, from (17) we have

$$ H=\frac{\Delta_{c} a(z)-a(z)}{g(z)} \bigl(e^{P(z+c)}-1 \bigr). $$

Note that $g(z)$ is an entire function. All the zeros of *H* come from the zeros of $\Delta_{c} a(z)-a(z)$ and $e^{P(z+c)}-1$. By $\nu_{F}(z)$ we denote the multiplicity of the zero of meromorphic function *F* at the point *z*.

Suppose that $z_{0}$ is a zero of *H*. We claim $\nu_{H}(z_{0})\leq \nu_{\Delta_{c} a-a}(z_{0})+\deg P\cdot \nu_{e^{Q}-1}(z_{0})$. Now we split into two cases.

*Case A.* $z_{0}$ is not a zero of $e^{P(z+c)}-1$. Then $z_{0}$ must be a zero of $\Delta_{c} a-a$. Hence $\nu_{H}(z_{0})\leq \nu_{\Delta_{c} a-a}(z_{0})$.

*Case B.* $z_{0}$ is a zero of $e^{P(z+c)}-1$. Set $P_{1}(z)=P(z+c)$. Obviously, $\nu_{e^{P_{1}}-1}(z_{0})\leq \deg P$. Then $H(z_{0})=0$ and $e^{P(z_{0}+c)}-1=0$, which leads to $e^{Q(z_{0})}=1$.

Assume that $e^{Q}\equiv 1$. Then we can rewrite (17) as

18 $$ \frac{\Delta_{c} a(z)-a(z)}{g(z)}=\frac{e^{P(z)}e^{P(z+c)}+e^{P(z+c)}-e ^{P(z)}-1}{e^{P(z+c)}-1}=e^{P(z)}+1. $$

Further,

19 $$ g(z)=\frac{\Delta_{c} a(z)-a(z)}{e^{P(z)}+1}, $$

we know $\Delta_{c} a(z)-a(z)$ is a small function of $e^{P(z)}$ and

$$ N \biggl(r,\frac{1}{e^{P(z)}+1} \biggr)=T \bigl(r, e^{P(z)} \bigr)+S \bigl(r, e^{P(z)} \bigr). $$

Thus, it follows from (19) that $g(z)$ is not an entire function, a contradiction. So $e^{Q(z)}\not \equiv 1$. The above discussion yields

$$\begin{aligned} \nu_{H}(z_{0}) \leq & \nu_{\Delta_{c} a-a}(z_{0})+ \nu_{e^{P_{1}}-1}(z _{0}) \\ \leq & \nu_{\Delta_{c} a-a}(z_{0})+\deg P \\ \leq& \nu_{\Delta_{c} a-a}(z _{0})+\deg P\cdot \nu_{e^{Q}-1}(z_{0}). \end{aligned}$$

Thus the claim holds.

By the claim and $T(r, e^{Q})=S(r, h)$, we get

$$\begin{aligned} N \biggl(r,\frac{1}{H} \biggr) \leq &N \biggl(r,\frac{1}{\Delta_{c} a(z)-a(z)} \biggr)+ \deg P \cdot N \biggl(r,\frac{1}{e^{Q}-1} \biggr) \\ \leq & T(r, \Delta_{c} a-a)+\deg P\cdot T \bigl(r, e^{Q} \bigr) \\ =& S(r,h). \end{aligned}$$

On the other hand, we have

$$\begin{aligned} 2T(r,h)&=T(r,H)+S(r,h) \\ &\leq \overline{N} \biggl(r,\frac{1}{H} \biggr)+\overline{N}(r,H)+ \overline{N} \biggl(r,\frac{1}{H-\alpha_{3}} \biggr)+S(r,h) \\ &\leq \overline{N} \biggl(r,\frac{1}{\alpha_{1}h^{2}+\alpha_{2}h} \biggr)+S(r,h) \\ &\leq T(r,h)+S(r,h), \end{aligned}$$

a contradiction. Thus, the case cannot occur.

Hence, we finish the proof of Theorem 1.

## Proof of Theorem 2

If $a(z)$ is a constant, then it follows from Theorem C that $f(z)\equiv \Delta_{c} f(z)$. In the following, we assume that $a(z)$ is a nonconstant entire function.

Suppose that $\Delta_{c}a(z)\equiv a(z)$. Then we have

$$ \frac{a(z+c)}{a(z)}=2. $$

Note that $\rho (a)<1$. Now, we apply Lemma 2.2 to this case.

Then there exists a *ϵ*-set *E* of finite logarithmic measure, so that

$$ \frac{a(z+c)}{a(z)}\rightarrow 1, $$

for all $z\rightarrow \infty$ in $\mathbb{C}\backslash E$. It is absurd. Thus, $\Delta_{c}a(z)\not \equiv a(z)$. It follows from (ii) of Theorem 1 that $\Delta_{c}f(z)=f(z)$ or

$$ \frac{\Delta^{2}_{c}f(z)-\Delta^{2}_{c}a(z)}{\Delta_{c}f(z)-\Delta _{c}a(z)}=\frac{\Delta^{2}_{c} a(z)-a(z)}{\Delta_{c} a(z)-a(z)}=e^{ \gamma }, $$

where *γ* is a polynomial.

Suppose that

$$ \frac{\Delta^{2}_{c}f(z)-\Delta^{2}_{c}a(z)}{\Delta_{c}f(z)-\Delta _{c}a(z)}=\frac{\Delta^{2}_{c} a(z)-a(z)}{\Delta_{c} a(z)-a(z)}=e^{ \gamma }. $$

Note that $\rho (a)<1$. We get

$$ \rho \bigl(e^{\gamma } \bigr)\leq \rho \biggl(\frac{\Delta^{2}_{c} a(z)-a(z)}{\Delta _{c} a(z)-a(z)} \biggr)\leq \rho (a) < 1, $$

which implies that $e^{\gamma }$ is a nonzero constant, say *C*. Furthermore, we get

$$ \Delta^{2}_{c} a(z)-a(z)=C \bigl(\Delta_{c} a(z)-a(z) \bigr). $$

Rewrite it as

$$ a(z+2c)-(2+C)a(z+c)+2C a(z)=0. $$

Applying Lemma 2.2 again, we deduce that $1-(2+C)+2C=0$, which implies that $C=1$. Then the above difference equation reduces to

$$ a(z+2c)-3a(z+c)+2a(z)=0. $$

Set $b(z)=a(z+c)-2a(z)$. Then the above equation can be rewritten as

$$ b(z+1)=b(z), $$

which implies that $b(z)$ is a periodic function. If $b(z)$ is a nonconstant function, then $\rho (b)\geq 1$. It contradicts $\rho (b)\leq \rho (a) <1$. Thus, *b* is a constant. Hence

20 $$ a(z+c)-2a(z)=b. $$

Then applying Lemma 2.2 again, there exists a *ϵ*-set *E* of finite logarithmic measure, so that

$$ \frac{a(z+c)}{a(z)}\rightarrow 1, $$

for all $z\rightarrow \infty$ in $\mathbb{C}\backslash E$. Rewrite (20) as

$$ \frac{a(z+c)}{a(z)}-2=\frac{b}{a(z)}. $$

Then choose a sequence $\{z_{k}\}$ such that $\vert z_{k} \vert =r_{k}$, $z_{k} \notin E$ and $\vert a(z_{k}) \vert =M(r_{k}, a)$, $r_{k}\rightarrow \infty $ as $k\rightarrow \infty $. Substituting $z_{k}$ into the above function yields a contradiction by Lemma 2.2. Thus, this case cannot occur. Then we deduce the desired result.

Hence, we finish the proof of Theorem 2.
